# Recent Progress in Spectroscopic Methods for the Detection of Foodborne Pathogenic Bacteria

**DOI:** 10.3390/bios12100869

**Published:** 2022-10-13

**Authors:** Mubashir Hussain, Jun Zou, He Zhang, Ru Zhang, Zhu Chen, Yongjun Tang

**Affiliations:** 1 School of Materials and Chemical Engineering, Hunan Institute of Engineering, Xiangtan 411104, China; 2 Postdoctoral Innovation Practice, Shenzhen Polytechnic, Liuxian Avenue, Nanshan District, Shenzhen 518055, China; 3 Hunan Key Laboratory of Biomedical Nanomaterials and Devices, Hunan University of Technology, Zhuzhou 412007, China

**Keywords:** pathogen detection, spectroscopy, biosensors, biomedical devices

## Abstract

Detection of foodborne pathogens at an early stage is very important to control food quality and improve medical response. Rapid detection of foodborne pathogens with high sensitivity and specificity is becoming an urgent requirement in health safety, medical diagnostics, environmental safety, and controlling food quality. Despite the existing bacterial detection methods being reliable and widely used, these methods are time-consuming, expensive, and cumbersome. Therefore, researchers are trying to find new methods by integrating spectroscopy techniques with artificial intelligence and advanced materials. Within this progress report, advances in the detection of foodborne pathogens using spectroscopy techniques are discussed. This paper presents an overview of the progress and application of spectroscopy techniques for the detection of foodborne pathogens, particularly new trends in the past few years, including surface-enhanced Raman spectroscopy, surface plasmon resonance, fluorescence spectroscopy, multiangle laser light scattering, and imaging analysis. In addition, the applications of artificial intelligence, microfluidics, smartphone-based techniques, and advanced materials related to spectroscopy for the detection of bacterial pathogens are discussed. Finally, we conclude and discuss possible research prospects in aspects of spectroscopy techniques for the identification and classification of pathogens.

## 1. Introduction

Foodborne pathogens cause diseases that affect both human health and the economy. Food and water are an essential part of life, and their contamination by bacteria poses a serious threat to human health and lifestyles [1,2]. Food-industry operators require rapid testing devices to monitor the quality of food for the presence of pathogenic bacteria [3]. Every year, millions of people worldwide get infected by contaminated food and water by microorganisms that cause various diseases. It is estimated that around 600 million foodborne diseases occur annually, with a mortality rate of 420,000 [4]. The Centers for Disease Control and Prevention estimated that approximately 2.5 billion people lack access to healthy and safe water in developing nations. Every year, more than 2.2 million mortality rates are reported due to waterborne diseases [5]. In China, a summary of studies estimated that the prevalence of pathogens in the food was 8.5% [6]. Controlling food safety is a persistent and challenging task in China due to the diversity of foods and food-production industries. Microbes such as *Escherichia coli*, *Staphylococcus aureus*, *Salmonella enterica*, and *Listeria monocytogenes* are usually highly infectious, and the presence of few colony-forming units (CFUs) can cause disease [7]. Therefore, it is crucial to identify pathogens at an initial stage with highly sensitive techniques to avoid diseases and outbreaks [8,9].

Conventional bacteria detection methods include cultivation, Gram staining, and biochemical analysis. These methods are reliable and have made great contributions to pathogen detection, but they are time-consuming and often take 2 to 3 days or more, which is not convenient for the rapid identification of microbes. The current techniques that are being used as clinical methods include polymerase chain reaction (PCR), enzyme-linked immunosorbent assay (ELISA), and matrix-assisted laser desorption ionization–time of flight (MALDI-TOF) mass spectrometry [10,11,12,13,14]. The latest techniques have revolutionized the field of diagnosis due to high sensitivity and specificity. However, some of the drawbacks associated with current clinical diagnostic techniques include high cost, controlled sampling conditions, being laborious and time-consuming, and the requirement for a skilled operator. Therefore, further research progress is required to develop a user-friendly, portable, and economic diagnostic development system. The development of simple techniques and systems for the rapid, economical, and on-site analytical approaches are essential in public health safety, medical diagnostics, and food safety [15,16,17,18,19].

This review is based on emerging detection approaches and methods based on spectroscopy for foodborne pathogens. Spectroscopy techniques have been used widely for the development of biomedical devices and prototypes. A large number of spectroscopy methods are emerging; however, this review focuses on recent progress in spectroscopic methods for pathogen detection, including surface-enhanced Raman spectroscopy, surface plasmon resonance, fluorescence spectroscopy, multiangle laser light scattering, and imaging analysis. Various bacteria show different spectroscopy characteristics, based on which the identification and classification of pathogens have been made. In the last few years, some trends in the area of spectrometry have emerged, including the use of nanoparticles, microfluidic platforms, specific biorecognition elements, and artificial intelligence [20,21]. Miniature devices related to spectroscopy microfluidic platforms in the field of biosensing have emerged in the last decade, and have been applied widely in biomedical devices [22,23,24]. Smartphones are ubiquitous, and user-friendly applications can be built for making point-of-care systems. The capability of smartphones for collecting and processing signals, images, data storage, and transmitting is suitable for creating miniature devices in remote and resource-limited areas. Recently, the integration of smartphones with other detection techniques has been emerging. The health-care applications of smartphones in biomedical devices and spectroscopy techniques provide portability and data-analysis feasibility, as well as economical and user-friendly systems. Instead of developing separate instruments and devices, smartphones are accessible to be utilized in many biomedical applications [25,26]. This review is organized to describe several spectroscopy techniques and approaches developed for the detection of pathogens to clinical diagnostics and food safety.

## 2. Surface-Enhanced Raman Spectroscopy (SERS)

Surface-enhanced Raman spectroscopy is a real-time detection method that depends on the inelastic scattering of excitation light and molecular resonance. SERS inherits the significant chemical fingerprint information on Raman spectroscopy and enhances sensitivity using plasmon-enhanced excitation and scattering. In SERS, the inelastic scattering from the molecules is greatly enhanced by a factor of up to 10^8^ when the molecules are adsorbed onto corrugated metal surfaces. The method can rapidly and efficiently detect a range of chemical structures and material compositions with high accuracy and reproducibility. These advantages make SERS a very promising tool for developing microbial detection techniques. SERS has been applied to different applications for detecting and classifying various pathogens [27,28,29,30,31].

Wang et al. developed a surface-enhanced Raman scattering (SERS)-based LFA strip for the detection of such pathogens as *Yersinia pestis*, *Francisella tularensis*, and *Bacillus anthracis*. Target-specific SERS nanotags (Raman reporter-labeled gold nanoparticles) were utilized instead of gold nanoparticles. The method detected the pathogens in a short duration of 15 min using a minimum sample volume of 40 µL. The obtained detection limits for *Y. pestis*, *F. tularensis*, and *B. anthracis* were 43.4 CFU/mL, 45.8 CFU/mL, and 357 CFU/mL, respectively [32]. A high-quality silver nanorod (AgNR)-based SERS substrate was prepared to acquire the chemical fingerprint information of 22 strains of common pathogens. The method was able to identify and discriminate 20 strains of pathogens (diluted to 10^7^ CFU/mL) with high sensitivity within 30 min [33]. Another SERS-based biosensor was fabricated using gold nanorods (GNRs) complexed with oligonucleotide aptamers. The SERS tags were combined with antibody-modified magnetic nanoparticles for the simultaneous detection of *Escherichia coli* and *Salmonella typhimurium*. The developed SERS biosensor showed a good linear response of 10^1^ to 10^6^ CFU/mL, high detection sensitivity (<8 CFU/mL) and a recovery rate of 95.26–107.88%. That study on combining aptamers and Raman reporters in SERS tags makes it possible to simultaneously detect different pathogens using a single biosensor [34].

Artificial intelligence has been applied widely in different diagnostics applications. Machine learning and neural networks are emerging techniques for data analysis and classification [35]. Spectroscopy data acquired from the SERS biosensors and techniques have been applied in machine learning and neural network algorithms. Ding et al. developed a method by combining SERS with a multiscale convolutional neural network (CNN). The label-free Raman substrate was prepared using gold nanoparticles. Different 1854 SERS spectra of three *Salmonella* serovars were measured and a multiscale CNN model was applied to extract SERS spectral features. The prepared gold nanoparticles and the developed CNN model showed detection accuracy higher than 97%. The given outcomes showed that the combination of SERS spectroscopy with multiscale CNN is feasible for *Salmonella* serotyping (*S. enteritidis*, *S. typhimurium*, and *S.* Paratyphi) with bacterial concentration of 10^8^ CFU/mL [36]. A stacked autoencoder-based deep neural networks algorithm was applied using SERS for the detection of methicillin-resistant *Staphylococcus aureus* and methicillin-sensitive *S. aureus.* The developed algorithm can evaluate features from the acquired signals and classify the data with an accuracy of 97.99%. The developed deep learning model classifies the pathogens with an area under the curve of 0.99 [37]. Ciloglu et al. combined SERS with machine learning techniques to classify *Staphylococcus aureus* and *Legionella pneumophila*. The technique gives higher classification accuracy of 97.8% by applying the *k*-nearest neighbors classifier [38].

Raman spectroscopy has been utilized extensively for microbiological diagnostics. A point-of-care testing technique has been developed using adhesive tape as a single platform for fast sampling, photocontrolled release, and SERS detection of pathogens from infected wounds. Pathogenic infections of *P. aeruginosa* and *S. aureus* were detected using gold nanostars on the adhesive tape as SERS substrate. The detection limit of the technique is 1.8 nM [39]. Duan et al. developed a SERS aptasensor for simultaneous detection of various pathogens using gold-decorated PDMS substrate. The fabricated film bound with the SERS probe to detect *Vibrio parahaemolyticus* and *Salmonella typhimurium* with a selectively detection limit of 18 CFU/mL and 27 CFU/mL, respectively [40]. The advancement of materials in SERS technology has increased accuracy and sensitivity for the detection of pathogens. The binding of SERS probes with fabricated chips and PDMS materials has enabled continuously miniaturization of detection prototypes [41,42].

Nakar et al. obtained spectra from pathogens *E. coli*, *Klebsiella pneumoniae*, and *Klebsiella oxytoca* isolates using UV-resonance Raman spectroscopy and single-cell Raman microspectroscopy. The obtained spectra were analyzed by machine learning algorithms for the classification of bacteria at the genus and species levels. The technique provides higher classification with 92% accuracy [43]. The method was further applied for the detection of clinical strains of *E. coli* [44]. Shen et al. created a fiber-probe-based method of Raman spectroscopy for the identification of six pathogens (*S. epidermidis*, *S. aureus*, *E. faecalis*, *E. faecium*, *P. aeruginosa*, and the yeast *C. albicans*). The collected signals from the fiber probe were analyzed using principal component analysis and linear discrimination models. The classification model acquired results with an accuracy of 93.8% [45]. The given studies were further extended and applied on agar plates to classify pathogenic infections [46].

**Figure 1:**
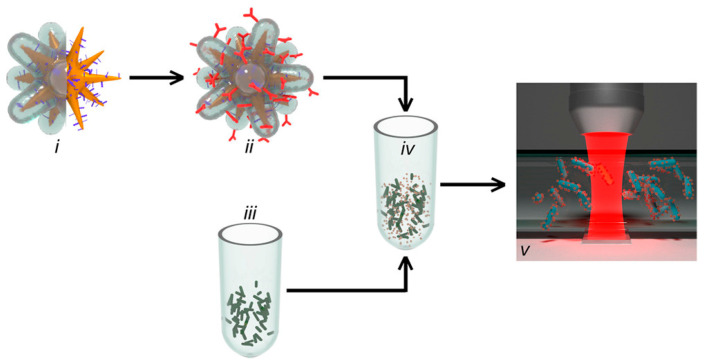
Schematic representation of the on-chip SERS technique for the detection of *L. monocytogenes*. (**i**) SERS-encoded gold nanostars. (**ii**) An antibody-binding protein. (**iii**) Test sample containing bacteria. (**iv**) Incubating bacterial testing sample with SERS tag. (**v**) Microfluidic channel for flowing the sample and SERS detection. Reprinted with permission from Ref. [47].

SERS scattering has been incorporated with a microfluidic chip for the identification and discrimination of pathogens using tagged gold nanostars. The testing sample flowed continuously through the microfluidic channel, and the SERS signal was acquired corresponding to the SERS-tagged nanostars coated with antibody-binding protein. The system is capable of discriminating between *L. monocytogenes* and *Listeria innocua* with a concentration of 10^5^ CFU/mL. Analyzing the data for the detection of pathogens requires less than 2 min. However, overall sample preparation and system operation time requires 30 min. Figure 1 is a schematic illustration of the on-chip detection of *L. monocytogenes* using SERS [47]. Bai et al. developed a sandwich immunoassay platform using functionalized SERS probes and magnetic beads for the simultaneous detection of *E. coli* and *S. aureus*. The technique uses two SERS probes for acquiring the signal following the immunomagnetic separation of the sample. The method identifies the pathogen with a detection limit of 10 and 25 CFU/mL for the simultaneous detection of *E. coli* and *S. aureus*, respectively [48]. Overall, SERS has emerged as a powerful analytical tool for rapidly detecting pathogens. Recent progress in the field of micro- to nanofabrication methodologies has enabled SERS applicable to various applications, such as rapid detection, point-of-care detection, and in situ detection. Currently, commercially available pathogen-detection techniques using SERS do not yet exist, but the improvement in SERS techniques has made it possible to develop handheld and portable prototypes to detect pathogens rapidly.

## 3. Surface Plasmon Resonance (SPR)

SPR is the fluctuation of the charge density at the interface between two media with dielectric constants of opposite signs, and the interaction between the media produces energetic plasmonic electrons. In SPR-based biosensors, the biological substance to be detected is immobilized on the sensor surface and the analyte typically passes through the sensor–analyte interface. The biorecognition event between the analyte and the biorecognition substance results in a change in the refractive index near the sensor surface, which is determined as a change in the plasmon resonance angle at the surface. SPR biosensors are used in many application areas because they are specific, sensitive, quantitative, and label-free analytical techniques [49,50,51,52,53].

Zhou et al. designed a fiberoptic surface plasmon resonance sensor based on antimicrobial peptides for the identification of *E. coli* O157:H7 in liquid medium. AgNP-rGO were coated on the optical surface and covered by gold film. The developed sensor had good specificity with a detection limit of 5 × 10^2^ CFU/mL [54]. Another biosensor based on optical fiber using SPR was designed for the detection of *E. coli.* The surface of U-shaped plastic optical fiber was immobilized with bacterial antibodies and coated with gold. The developed biosensor is economical, rapid, and showed a detection limit of 1.5 × 10^3^ CFU/mL [55].

A highly sensitive SPR biosensor consists of the prism, gold coating, graphene, affinity layer, and sensing medium for the detection of waterborne pathogens. The structural parameters of the biosensor were optimized to attain a higher sensitivity of 221.63°/RIU for *E. coli* and 178.12°/RIU for *Vibrio cholera* pathogen with an average value of 199.87°/RIU [56]. An optical sensor based on thin liquid film was designed by combining the SPR, light extinction, and near-critical angle reflection. The calculated sensitivity of SPR to the surface refractive index was 168.35°/RIU. The experiments were performed to evaluate the reflectivity curve from the sample containing *E. coli* at a concentration of 4.7 × 10^8^ CFU/mL [57]. Another SPR biosensor was designed using flexible photonic crystal fibers. A metallic gold strip and titanium oxide film were coated on the outer surface of the biosensor. The simulation results showed higher amplitude sensitivity of 7420.69 RIU^−1^ and wavelength sensitivity of 87,000 nm/RIU. The given technique requires real-time implementation for the detection of different biological materials [58]. The surface plasmon resonance imaging enabled crossed surface relief gratings utilized for the rapid and label-free detection of *E. coli*. The prototype was connected with optics and electronics systems. The testing was performed using clinical samples within the concentration range of 10^3^–10^9^ CFU/mL. The acquired detection limit of the system was approximately 100 CFU/mL, which is below the threshold value for clinical urinary tract infection diagnosis [59].

**Figure 2:**
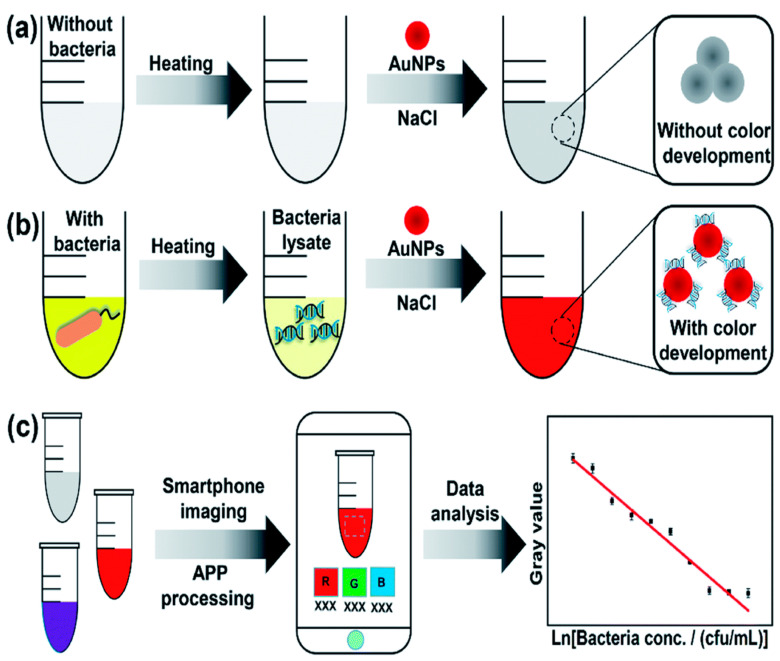
Schematic demonstration of the smartphone-based SERS platform for the classification of bacterial samples. (**a**,**b**) Testing samples with and without bacteria mixed with AuNPs. (**c**) The acquired RGB signal of the captured images was utilized for detecting the bacterial concentrations. Reprinted with permission from Ref. [60].

Wen et al. presented a smartphone-based SPR sensing platform for the fast identification of *E. coli*. The SPR phenomena of gold nanoparticles were used for pathogen sensing. The smartphone was used for sensing the signal dependent on AuNP color variations. The image-processing technique was applied to evaluate the spectral color intensity of the bacterial sample in response to SPR. The proposed technique requires less detection time without using complicated laboratory instrumentation. The detection limit of the developed method is 8.81 × 10^4^ CFU/mL [60]. A schematic illustration of the developed system is presented in Figure 2. Hence, recent research shows the synthesis of different nanomaterials, and applications in SPR exhibit excellent efficiency for pathogen detection. Each nanomaterial with different 3D structure has its own merits with distinctive optical characteristics and several reaction patterns to analytes.

## 4. Fluorescence Spectroscopy

There are various fluorescence spectroscopy techniques for direct and indirect identification of foodborne pathogens. Conventional fluorescence spectroscopy relies on organic dye-labeled recognition probes. Recently, advances in the development of various materials, such as quantum dots, metal–organic frameworks, polymers, and carbon dots, have been used as fluorescence tags in assays for the detection of pathogens [61,62,63,64,65]. Optical transducers based on advanced development of materials are particularly attractive for the rapid and direct detection of pathogens. Direct fluorescence techniques are based on utilizing naturally fluorescent components that have been utilized for bacterial identification. Different reactions of certain enzymes with the cells emit photons as a byproduct. The emission of photons during the reaction creates fluorescence that is utilized in the detection process of various pathogens [66,67,68,69,70].

**Figure 3:**
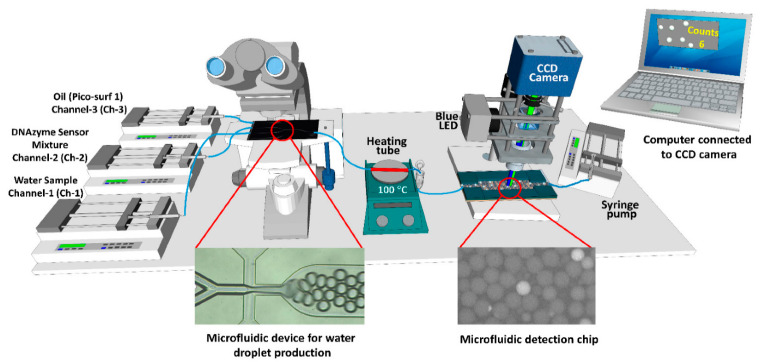
Schematic representation of the microfluidic-based device for the isolation and detection of *E. coli* using fluorescence detection. Reprinted with permission from Ref. [74].

Zhao et al. developed a highly sensitive immunosensor for the rapid identification of *E. coli* using microspheres labeled with carbon dots. Fluorescence spectroscopy was applied to analyze the emission of excitation wavelength. The developed immunosensor has a detection limit of 2.4 × 10^2^ CFU/mL in milk and can be tested within 30 min [71]. A highly sensitive biosensor was developed using a terbium-based metal–organic framework interfaced with anti-*E. coli* antibodies. The biosensor is capable of detecting *E. coli* in analytes within the range of 1.3 × 10^2^ to 1.3 × 10^8^ CFU/mL with a detection limit of 3 CFU/mL. The total time to perform the detection experiment is about 20–25 min, with a response time of 5 min [72]. Kim et al. developed a microfluidic nanobiosensor for the detection of *Salmonella* using quantum dot nanoparticles. A miniature fluorometer was designed to detect the fluorescence signal from the quantum dot nanoparticles linked with *Salmonella.* The fluorescence detection module was coupled with fibers for the transmission of the optical signal. The sensor is capable of detecting microbes with a limit of detection of 10^3^ CFU/mL in both buffer solution and food samples [73]. Rauf et al. designed a digital counter to isolate and detect *E. coli* from the water using a microfluidic platform and computer vision. The droplets were generated using sample in water and DNAzyme. The DNAzyme creates fluorescence in the presence of *E. coli*, and the generated fluorescence was used for the detection of pathogens. The generated droplets were incubated in a heating tube and then passed to the microfluidic detection chip. The droplets containing *E. coli* exhibit fluorescence that was analyzed using computer-vision based algorithm. The detection process can be performed using a minimum volume of 50 µL. The system can detect pathogens with 100 cells in a volume of 50 µL. The overall scheme of the developed prototype is presented in Figure 3 [74].

Droplet incubation is emerging for the development of rapid diagnostic methods. Kaushik et al. presented a DropFast technique using a rapid resazurin-based fluorescence to cultivate *E. coli* inside a picoliter droplet. The pathogens encapsulated inside the 20 pL droplets were incubated for an hour, and the fluorescence detection method analyzed the antimicrobial sensitivity. The detection experiments were performed with a sample concentration of 10^7^ CFU/mL [75]. Another proof-of-concept study was performed to detect pathogenic DNA using multiple loop-mediated isothermal amplification (LAMP). The microfluidic chip detected three pathogens: *E. coli*, methicillin-resistant *S. aureus*, and methicillin-sensitive *S. aureus*. The testing procedure was performed within 2 h, and the detection limit of the specific genes was less than 10^2^ CFU/100 mL [76]. A gel-based loop-mediated isothermal amplification (gLAMP) integrated with a microfluidic chip for the detection of different pathogens has been tested. Microchannels allow DNA samples to flow to the reaction chamber in the chip. The fluorescence imaging system was used to analyze the sample. The system detected pathogens with high selectivity and sensitivity of fewer than 1.6 cells. The pathogen mixture was detected simultaneously with 96 copies of *P. hauseri* and 36 copies of *Salmonella*. *E. coli* was detected using 35 copies [77]. Huang et al. developed a portable microfluidic chip-based nucleic acid analyzer for the detection of *Mycoplasma pneumoniae*, *Staphylococcus aureus*, and *methicillin-resistant S. aureus.* A portable nucleic acid analyzer was developed for analyzing the fluorescence data of nucleic acid amplification in real time. The device detected extremely low DNA concentration with a detection limit of 10^1^ copies/µL with high sensitivity and accuracy. The overall required time duration from sample preparation to detection results requires less than 90 min [78]. Chen et al. developed a portable multichannel turbidity system for the rapid identification of pathogens using LAMP. The developed system consists of a temperature controller, photoelectric detection system, and calibration system. The designed system is capable of detecting *Legionella* bacteria and H7 subtype virus (H7) within 1 hour. The system is more specific for *Legionella* bacteria, with sensitivity for H7 of 10 copies/mL [79].

Wang et al. developed a smartphone-integrated paper sensing system using fluorescent and colorimetric dual readout for the detection of *E. coli.* The presence of pathogen changes the fluorescence and the UV-vis absorbance signals. The variation in the fluorescence is detected by the developed smartphone application for color scanning. The designed technique showed good sensitivity with a detection limit of 100 CFU/mL and 44 CFU/mL by fluorescence and colorimetric assay, respectively [80]. Smartphone-based microscopes have been developed for various applications in medical diagnosis and pathogen detection. An optimized peptide nucleic acid (PNA)-based fluorescence in situ hybridization (FISH) assay was used with a smart-phone based fluorescence microscope. The designed system is capable of detecting pathogenic *Cronobacter* spp. with a limit of detection of 10^4^ CFU/mL [81]. Overall, fluorescence-based biosensors are highly sensitive and with a wide dynamic range that enables the rapid detection of pathogens. The development of nanoparticles as fluorescence probes to enhance fluorescence intensity in the presence of biological samples have shown advantages in the rapid detection of pathogens.

## 5. Multiangle Laser Light Scattering

Lasers are widely used for the detection of microorganisms because of high-intensity and monochromatic features. Various light-scattering theories, including Rayleigh theory, Mie scattering, and Rayleigh–Gans theory, have been applied to predict homogeneous particles [82,83,84,85,86]. Modern devices based on light-scattering techniques are designed based on mathematical and physics-related models. Dynamic light scattering (DLS) is based on the principle of Brownian movement and analyzes the temporal fluctuations of the scattered light intensity. DLS has been applied widely to estimate the size of particles from the scattered light in an aqueous medium and for the detection of biological samples. Pathogen detection has been carried out using DLS, in which the pathogens are considered microparticles. Different pathogens exhibit unique scattering of light based on different sizes, shapes, and characteristics of the microbes [87,88,89,90,91].

Hussain et al. built a prototype for sensing the scattered laser light from microbes. The prototype was designed based on the MIE scattering theorem, which gives useful information about the scattering of light from particles. The prototype consists of an assembly of 32 photosensors, laser light, and a data-acquisition system. The optimum concentration of the sample was used, and the laser light passed by the prepared sample. The surrounding photodetectors captured the scattering of light, and the data were analyzed using statistical analysis for the classification of pathogens. *E. faecalis*, *S. aureus* and *E. coli* microbes were tested with 50–70 cells in 10 mL DI water. The mean classification accuracy for *E. faecalis*, *S. aureus,* and *E. coli* was 81.8%, 70.9%, and 71.4%, respectively [92]. The prototype consists of an assembly of 32 photosensors, placed at different positions surrounding the sample flask. The higher number of sensors requires greater computing power, which reduces the efficiency of the system and takes more time for processing data [93]. Therefore, the prototype was further modified by reducing the number of surrounding sensors using MIE scattering. MIE scattering theory states that higher intensity of scattering occurs in the forward direction compared to back- and side scattering. Therefore, the photosensors placed in the back direction were removed and the number of sensors reduce to 12 [84]. A machine learning algorithm was applied to the acquired data. The device classified the pathogens *E. faecalis*, *E. coli,* and *S. aureus* with an accuracy of 99%, 87%, and 94%, respectively. The overall classification accuracy of the machine learning model was 93.6% [94]. The technique can detect and identify pathogens with 50–60 microbes in a volume of 10 mL. The designed prototype was further extended and applied for the detection of hepatitis B surface antigen (HBsAg) based on immunomagnetic separation. The results showed classification accuracy for the identification of HBsAg was 87.7%, with a dynamic range of 98.86 IU/mL to 3163.5 IU/mL [95].

**Figure 4:**
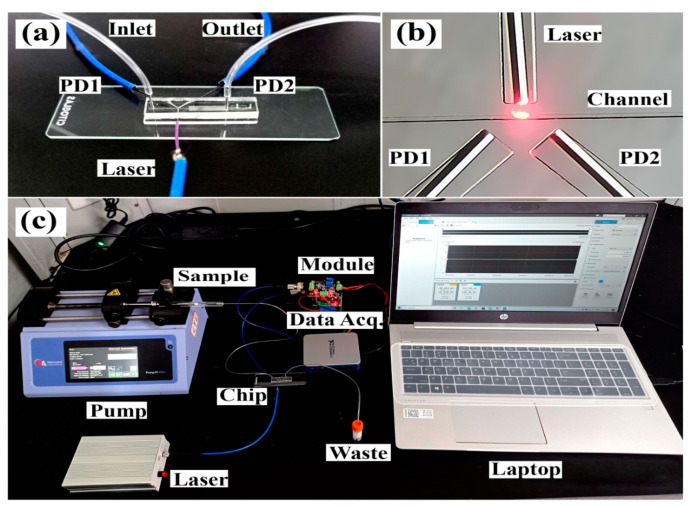
(**a**) Fabricated microfluidic chip for passing the laser light and collecting the scattered light. (**b**) Microscopic view of the microfluidic channel for passing pathogenic sample. (**c**) A prototype connected with laptop for the identification of microbes using the principle of scattered light signals from the microfluidic platform. Reprinted with permission from Ref. [98].

The microfluidic platform was designed for the identification of pathogens using the scattering of light from microbial particles. Microfluidic devices are desirable due to miniaturization, portability, and requiring less sample volume. The controlled continuous flow of microbes from the microfluidic channel helps in detecting the scattered light. Microfluidic chips are playing an essential role in the advancement of POCT devices [96,97]. An embedded microfluidic chip platform has been linked with optical fibers for connecting photosensors and laser light. The pathogenic sample was separated using immunomagnetic separation, and separated magnetic beads flowed through the microfluidic channel. The laser light passed through the microfluidic channel and the photosensors collected the scattered light from the flowing magnetic beads. Figure 4 describes the developed microfluidic chip platform and prototype for detecting scattered light from the magnetic beads. The scattered light was classified using machine learning algorithms. Higher classification accuracy of 97.9% was acquired for the detection of *P. aeruginosa* with a detection limit of 10^2^ CFU/mL. The device can perform the detection procedure within 25 min [98].

## 6. Imaging Analysis

Image processing and analysis are extensively applied for the classification of biological substances. Extensive research has been carried out in developing fluorescence tags to be utilized in imaging techniques for microbial classification [99,100,101,102]. Microscopy techniques and miniature smartphone-based detection devices are developed for collecting and classifying the images for the detection of pathogens. Classification algorithms are applied widely in many applications of biomedical imaging analysis and classification [103,104,105,106]. Deep diagnostic agent forest is a deep-learning pathogen-recognition system proposed for the detection of pneumonia using CT images. The deep-learning algorithm shows higher classification accuracy for pneumonia pathogen recognition using CTs [107]. A combination of fluorescence imaging and deep-learning automated identification of the fecal contamination on meat has been applied. The developed efficient deep-learning model achieved 97.32% accuracy and specificity of 97.35% for discriminating between clean and contaminated areas on meat [108].

**Figure 5:**
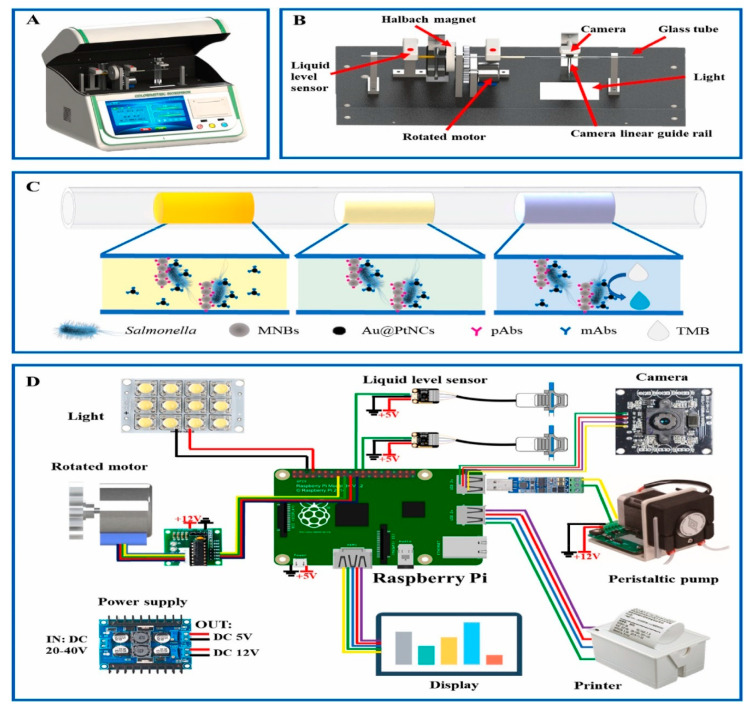
(**A**) The developed prototype of the lab-on-a-tube biosensor. (**B**) The internal view of the operating device. (**C**) The modified glass tube uses various magnetic beads, particles, and biological materials. (**D**) Various components of the electrical hardware. Reprinted with permission from Ref. [110].

A miniature system was developed using smartphone-based lateral-flow imaging and machine learning for detecting *Salmonella* spp., with a detection limit of 5 × 10^4^ CFU/mL. An optical imaging system was optimized with an angled slot to enhance the optical intensity. The device gives classification accuracy of 95.56% using the combination of RGB color space and machine learning classifiers [109]. Qi et al. developed an automated and portable system for detecting pathogens using rotated Halbach magnetic separation and Raspberry Pi imaging. The prepared magnetic nanobeads captured the targeted pathogens, and the captured images were analyzed to quantitively determine the concentration of pathogens. The developed system was able to detect *Salmonella* with a detection limit of 8 CFU/50 μL in 60 min. Figure 5 shows representative images of the developed prototype and methodology [110].

Imaging analysis for droplets encapsulated with pathogens has been applied in various research areas for detecting biological materials. Specifically, droplet-based bioreactors are widely applied for incubating pathogens and detection. Zhu et al. developed a microfluidic technology to analyze the quantitative growth of *Bacillus coagulans.* The generated droplets were encapsulated with microbes and then incubated to grow the cells in the droplets. The incubated droplets were analyzed using microscopic fluorescence images. The growth of the *B. coagulans* cells was estimated by bright-field images and fluorescence intensity in the droplets. The microbial growth in the droplets showed good consistency, with a correlation coefficient of 0.98 [111]. Another droplet incubation-based system was designed for accurate diagnosis of antibiotic-resistant gut microbes. The incubated droplets were reinjected into the microfluidic chip, and images were collected through a high-frame-rate camera on the microscope. The growth of microbes was analyzed by imaging analysis, represented by wavelet optical density value [112]. A portable microfluidic chip has been designed for the detection of *Salmonella* based on single-cell droplets. The generated droplets were cultivated with resazurin that produced fluorescence of the cultivated droplets. The fluorescence from the cultivated droplets was used to distinguish the sample within 5 h. The system can detect pathogens with a detection limit of 50 CFU/mL [113]. Kim et al. proposed a microscopy-based framework for the rapid identification of pathogens from single to a few cells. The technique obtains and utilizes the morphology of testing samples by incorporating 3D quantitative phase imaging and an artificial neural network. The system identified 19 bacterial species with a classification accuracy of 82.5% from a specific bacterial cell or cluster [114].

Imaging analysis and classification using artificial intelligence have significantly enhanced classification accuracy. Data deep learning and neural networks have made classification more rapid compared to machine learning techniques that use feature acquisition. AI coupled with spectroscopy techniques could bring significant advances in the field of biological detection techniques, but there are more challenges in selecting an appropriate technique to be applied for a specific problem. In addition, the extensive quantity of clinical data is demanding in terms of validating the results and applying methods at commercial level. Analysis of SERS, SPR, fluorescence spectroscopy, and multiangle laser light scattering for the detection of pathogens has been summarized in Table 1.

## 7. Conclusions and Future Perspectives

This paper provides an overview of the application of spectroscopy techniques for developing foodborne pathogen-detection methods over recent years. The consumption of pathogen-contaminated food and water poses a serious threat to human life. The rapid and accurate identification of pathogens can avoid epidemics of severe foodborne diseases. The latest spectroscopy techniques incorporate miniature and POCT devices. The progress in the material sciences and fabrication techniques has made it possible to manufacture miniature optical instruments. The synthesis of advanced materials and fabrication of nanoparticles on SERS substrates has improved the SERS detection of various foodborne pathogens. Further research will be required in the characterization of materials to enhance SERS detection and improve sensitivity and selectivity.

Artificial intelligence techniques, including machine learning and deep learning, have been applied widely for data classification. For analyzing spectroscopy data, classification models based on machine learning have shortened the time required for high classification accuracy compared to using statistical analysis and mathematical models. The advent of deep learning and neural networks has significantly increased the classification accuracies in data classification. Deep learning, microfluidics, advanced materials, and robotics will enable automation and high throughput in pathogenic diagnostics.

Advancements in instrumentation have enabled the creation of portable and easy-to-assemble devices. Microfluidic chips are also capable of being integrated with laser light, photosensors, and Raman scattering detectors. The smartphone has been utilized widely for the classification of pathogenic microbes using microfluidics, imaging techniques, and detecting fluorescence. Smartphones have built-in high-definition cameras utilized for microscopy and fluorescence detection. The internal microprocessors of a smartphone can acquire image and signal processing without external computers and share real-time outcomes speedily. Smartphone application development is user-friendly and easy to access for users, and the acquired data can be linked to servers for data processing. Automated microfluidic platforms are capable of sample processing, separation, droplet generation, and incubation. All these capabilities can be utilized to develop smart devices using the spectroscopy principle. Meanwhile, significant challenges, including label-free detection, shortening sample-preparation methods, system portability, cost, and rapid detection, still need to be addressed in developing spectroscopy techniques to be applied at a commercial level.

## Figures and Tables

**Table 1:** Different spectroscopy techniques for the detection of foodborne pathogens. The information about the available literature of the given methods is included in the main text.

Detection Technique	Detecting Pathogens	Performance	Detection Limit	Ref.
**Surface-Enhanced Raman Spectroscopy (SERS)**				
LFA strip-based SERS	*Y. pestis*, *F. tularensis*, and *B. anthracis*	40 µL testing sample, assay time 15 min	*Y. pestis* 43.4 CFU/mL, *F. tularensis* 45.8 CFU/mL, and *B. anthracis* 357 CFU/mL.	[32]
AgNR based SERS	20 strains of pathogens	Discriminate 20 strains of pathogens, detection time 30 min	10^7^ CFU/mL	[33]
GNRs based SERS	*E. coli* and *S. typhimurium*	Simultaneous detection, linear response, recovery rate 95.26–107.88%	<8 CFU/mL	[34]
SERS using CNN	*S. enteritidis, S. typhimurium,* and *S.* Paratyphi	Label-free Raman substrate, Classification accuracy 97%	10^8^ CFU/mL	[36]
SERS using DNN	methicillin-resistant *S. aureus* and methicillin-sensitive *S. aureus*	Label-free SERS, classification accuracy 97.99%	-	[37]
SERS using ML	*S. aureus* and *L. pneumophila*	Discriminate antibiotic-resistant bacteria, classification accuracy 97.8%	-	[38]
SERS Adhesive Tape	*P. aeruginosa and S. aureus*	POC testing, Rapid detection, detection process 8 h	1.8 nM	[39]
SERS aptasensor using gold decorated PDMS substrate	*V. parahaemolyticus* and *S. typhimurium*	non-overlapping Raman peaks, low cost, simultaneous detection	*V. parahaemolyticus* 18 CFU/mL and *S. typhimurium* 27 CFU/mL	[40]
Machine learning spectra analysis	*E. coli, K. pneumoniae* and *K. oxytoca* isolates	Label free, classification accuracy 92%	-	[43]
Fiber-probe-based Raman Spectroscopy	*S. epidermidis*, *S. aureus*, *E. faecalis*, *E. faecium*, *P. aeruginosa*, and the yeast *C. albicans*	Rapid, portable strategy, accuracy 93.8%	-	[45]
SERS tags with microfluidic	*L. monocytogenes* and *L. innocua*	Real-time detection, total analysis time 30 min.	10^5^ CFU/mL	[47]
Immunoassay platform	*E. coli* and *S. aureus*	Simultaneous detection, highly sensitive and selective technique	*E. coli* 10 CFU/mL and *S. aureus* 25 CFU/mL	[48]
**Surface plasmon resonance (SPR)**				
Fiber optic-based SPR	*E. coli*	Recovery rate of 88%~110%, high specificity	5.0 × 10^2^ CFU/mL	[54]
Fiber optic-based SPR	*E. coli*	Selective, portable system, economical and rapid	1.5 × 10^3^ CFU/mL	[55]
SPR (prism, gold coating, graphene, affinity layer)	*E. coli* and *V. cholera*	Higher sensitivity: 221.63°/RIU for *E. coli* and 178.12°/RIU for *Vibrio cholera*	-	[56]
SPR based on the thin liquid film	*E. coli*	Economical, label free, rapid, Higher sensitivity: 168.35°/RIU, minimum sample volume ≈10 μL	4.7 × 10^8^ CFU/mL	[57]
SPR imaging	*E. coli*	Rapid, label-free detection, economical system design (∼US$100) and detection time (35 min)	~100 CFU/mL	[59]
Smartphone-based SPR	*E. coli*	Real-time detection, equipment-free assay, and POC detection	8.81 × 10^4^ CFU/mL	[60]
**Fluorescence Spectroscopy**				
Microspheres labeled with carbon dots	*E. coli*	Higher sensitivity, detection time 30 min	2.4 × 10^2^ CFU/mL	[71]
Terbium-based metal organic framework	*E. coli*	Experiment time 20–25 min, response time 5 min	3 CFU/mL	[72]
Fluorometer using quantum dot nano-particles	*Salmonella*	Microfluidic platform, miniature device	10^3^ CFU/mL	[73]
Digital counter using a microfluidic platform	*E. coli*	Microfluidic platform, 50 µL testing sample	100 cells in a volume of 50 µL	[74]
Rapid resazurin-based fluorescence	*E. coli*	20 pL droplets incubation, antimicrobial sensitive method, detection time 1 h	10^7^ CFU/mL	[75]
LAMP	*E. coli, methicillin-resistant S. aureus and methicillin-sensitive S. aureus*	Detection within 2 h	10^2^ CFU/100 ml	[76]
gLAMP integrated with a microfluidic chip	*P. hauseri*, *Salmonella*, and *E. coli*	Simultaneous detection, high selectivity and sensitivity of fewer than 1.6 cells	*P. hauseri* 96 copies, *Salmonella* 36 copies, and *E. coli* 35 copies	[77]
Microfluidic chip-based nucleic acid analyzer	*M. pneumoniae*, *S. aureus*, and *methicillin-resistant S. aureus*	Portable system, Detect low DNA concentration, detection less than 90 min	10^1^ copies/µL	[78]
multichannel turbidity system using LAMP	*Legionella* bacteria and H7 subtype virus	Rapid detection within one hour	10 copies/mL	[79]
Smartphone-integrated paper sensing system using fluorescence	*E. coli*	Smartphone application, user-friendly system	100 CFU/mL	[80]
Smartphone-integrated paper sensing system using colorimetric dual readout	*E. coli*	Smartphone application, user-friendly system	44 CFU/mL	[80]
Smartphone-based microscope	*Cronobacter spp.*	Miniature device, optimized PNA-based FISH assay	10^4^ CFU/mL	[81]
**Imaging Analysis**				
Fluorescence imaging and deep learning	*E. coli* and *Salmonella*	Classification accuracy 97.32%,specificity 97.35%	-	[108]
smartphone-based lateral-flow imaging and machine learning	*Salmonella spp.*	Classification accuracy 95.56%	5 × 10^4^ CFU/mL	[109]
Halbach magnetic separation and Raspberry Pi imaging	*Salmonella*	Automated detection device, operation time 1 h, recovery rate from 88.96% to 99.74%	8 CFU/50 μL	[110]
Incubated droplets imaging	*B. coagulans*	Correlation coefficient 0.98	Droplet seeding density approx. 9 × 10^7^ cells/mL	[111]
Droplets imaging using resazurin	*Salmonella*	Single-cell detection, testing within 5 h	50 CFU/mL	[113]
Microscopy-based framework	19 bacterial species	Classification accuracy of 82.5%	Single to several cells and over 10^5^ CFU	[114]
